# Efficient and accurate personalized product recommendations through frequent item set mining fusion algorithm

**DOI:** 10.1016/j.heliyon.2024.e25044

**Published:** 2024-01-19

**Authors:** Lifeng Kang, Yankun Wang

**Affiliations:** Jiaozuo Normal College, Jiaozuo, 454000, China

**Keywords:** Fusion recommendation algorithm, Frequent item set mining

## Abstract

In the realm of personalized product recommendation, addressing the challenges of sparse data and “cold start” has been the primary focus. However, filtering invalid information amidst the overwhelming data on e-commerce platforms remains an underexplored issue. This paper proposes a fusion recommendation algorithm based on frequent item set mining to tackle this problem by compressing the commodity data set and identifying the frequent commodity set. The algorithm not only improves time efficiency by reducing the number of candidate frequent item sets but also generates more accurate recommendations by calculating user-commodity interest rankings and recommending similar products. We first present the existing problems in fusion recommendation algorithms based on frequent item set mining, such as redundant rules, low recommendation accuracy, and the inability to explore deep connections between users and products. Next, we introduce our proposed algorithm, which involves filtering the commodity data set, calculating user-commodity interest rankings, and defining similar product recommendation rules. The algorithm's effectiveness is demonstrated by its ability to adapt to users' dynamic preferences and capture their changing interests in real-time. A comparative analysis using our algorithm and other data mining algorithms reveals a reduction in the number of frequent commodity data sets and weighted frequent item sets, leading to decreased algorithm operation time. This research contributes to the development of more efficient and accurate personalized product recommendation algorithms, enhancing user experience on e-commerce platforms.

## 1 Introduction

Electronic commerce is a commercial activity that adopts information network technology and conducts business through network and electronic transactions. Due to the convenience of e-commerce trading, the trading field is more extensive and presents a state of globalization. Compared with the traditional offline sales mode, e-commerce breaks through the limitations of time and space and has the advantages of low cost, broad scope and high efficiency [1,2]. The primary forms of e-commerce are online shopping, online electronic payment and online business communication and transaction, and enterprise financing and comprehensive service business have gradually become a new way of operation [3]. With the rapid development of e-commerce, e-commerce activities are increasing worldwide. Many Internet companies, such as Alibaba, Amazon, Jingdong, etc., have created an e-commerce-centred business system [4]. E-commerce website usability analysis has been an essential factor in enhancing user experience, and researchers have explored association rule mining and machine learning algorithms to improve it [5].

The online and offline one-stop service has attracted more consumers, enhanced the data support of the platform, and formed a virtuous circle between consumers and the platform through data analysis and feedback. At the same time, the considerable income of the e-commerce platform has also attracted many investors and service providers to join, and the competition is becoming increasingly fierce. How to find new avenues and opportunities based on e-commerce platform services has become another new trend in the development of e-commerce [6].

The further development of Internet technology and the continuous expansion of the e-commerce market have brought consumers a wide variety of products so that consumers have more choices than before. However, the resulting problem is that the quantity of goods has reached an unprecedented scale, which makes consumers unable to choose the goods they need within a limited time quickly [7]. The selection of goods takes a long time, and the results are not always satisfactory. In today's fast-paced life, low-efficiency online shopping must be unbearable. The emergence of the recommendation system solves this problem well [8]. As a supplement to the search engine, the recommendation system can significantly improve the shopping efficiency of consumers, effectively explore users' interests and provide personalized recommendations [9]. It can improve the transformation of goods and the utilization rate of commodity resources by integrating the recommendation system into the e-commerce platform. All the commodities can be reorganized and optimized by analyzing the historical search and purchase information of consumers on the platform. It can also fully understand the current demands of consumers. Through personalized recommendations and customized displays, a good shopping experience is provided for consumers, which effectively saves consumers' time and improves shopping efficiency [10]. The e-commerce platform also achieves the purpose of increasing commodity sales. The product recommendation algorithm is an essential link in electronic commerce. According to the user's preferences, the recommendation system will put the goods that the user is more likely to buy in the front position and the goods that the user is less interested in the back position. The list of goods users sees in the mall is sorted by the recommendation algorithm and presented in order.

However, the performance of the recommendation algorithm in the current recommendation system is not satisfactory in understanding user operations. Among them, the system still recommends similar products after the user purchase operation, especially the problem [11]. Usually, after the purchase operation, the user will have the psychological expectation of owning the product, the demand is satisfied, and will not repurchase the same product. At this time, the recommendation of the same product will not only make the recommendation function fail to play a role but also reduce the user experience [12]. It may be the most straightforward solution to directly regarding the purchase behaviour as having a negative impact on the degree of interest in the product. However, if the users with the habit of repeated purchases and the products that need to be repeatedly purchased cannot be excluded, this approach will reduce the user experience of other aspects of the recommendation system [13].

This paper presents a fusion recommendation algorithm based on frequent item set mining to improve recommendation efficiency and accuracy in the e-commerce industry. This research makes several notable contributions to the field of personalized product recommendation and information filtering. We introduce a novel fusion recommendation algorithm that effectively addresses the issues of information overload, data sparsity, and cold start in existing recommendation systems. Our method incorporates a user-commodity interest ranking calculation, which considers multiple user behaviors, allowing for more accurate predictions of user preferences and a more personalized recommendation experience. Furthermore, we present a new approach for calculating the similarity of similar products, incorporating multi-level classifications and various attributes, which helps to provide more relevant recommendations to users. We also provide a thorough comparative analysis of our proposed algorithm against traditional recommendation methods, demonstrating the improved efficiency, effectiveness, and recommendation quality of our approach.

By highlighting these contributions, we hope to emphasize the significance and impact of our research in addressing the challenges faced by personalized product recommendation systems. In the following sections, we will discuss the existing problems in fusion recommendation algorithms, present our algorithm design, and provide a detailed analysis of the results obtained from our experimental evaluation.

The paper is organized into five sections: (1) Introduction, which provides context and highlights the challenges in recommendation systems; (2) Literature Review, where existing research and algorithms are discussed; (3) Fusion Recommendation Algorithm Based on Frequent Item Set Mining, detailing the proposed algorithm and its potential advantages; (4) Experimental Results and Analysis, evaluating the performance of the algorithm through experiments and comparisons with existing algorithms; and (5) Conclusion, summarizing the findings and suggesting future research directions.

## 2 Literature review

Recommendation systems have gradually developed alongside advancements in internet technology. The concept first appeared in the 1990s when the GroupLens research group at the University of Minnesota designed a news recommendation system called GroupLens. This work introduced the idea of collaborative filtering and established a formal model for the recommendation problem, significantly impacting the development of recommendation systems in the subsequent decades [14]. The research group went on to create the MovieLens recommendation website, an academic platform for studying recommendation engines that now contains one of the most widely-used datasets in the recommendation field. Recommender Systems were first proposed by Paul Resnick and Hal R. Varian in 1997 and have since been widely cited [15]. Many enterprises have recognized the potential of recommendation systems to boost sales and have invested heavily in research, making recommendation systems a hot research topic.

In 1998, Amazon launched a project-based collaborative filtering algorithm and applied it to e-commerce, yielding positive recommendation results. In 2003, Linden et al. of Amazon published a paper announcing the project-based collaborative filtering algorithm. According to statistics, Amazon's revenue increased by 30 % due to the research and application of the recommendation system [16]. In March 2007, Google added personalized elements to the AdWords monetization model, allowing for better analysis of users' preferences and needs, improving the quality of advertising recommendations, and increasing profits [17]. In recent years, the development of big data and other technologies has led to the widespread use of recommendation systems in e-commerce, music, news, personalized advertising, and other fields, achieving remarkable results [18].

The current research on recommendation systems mainly focuses on two aspects: the research and optimization of recommendation algorithms and the engineering application of recommendation algorithms. Commonly used recommendation algorithms include content-based recommendation algorithms [19], collaborative filtering recommendation algorithms [20], and hybrid recommendation algorithms [21]. The optimized content usually addresses issues such as data sparsity, cold start, and large volumes of data in the recommendation process. Data sparsity is reflected in the score matrix, where the number of user score data is minimal compared to the number of items, resulting in suboptimal recommendation effects. The cold start problem arises when a recommendation system encounters new users, making it challenging to generate recommendation results due to the lack of relevant data for new users. The problem of large amounts of data is manifested in the vast scale of user and project data, making it difficult for the recommendation system to process and provide recommendation results in a timely manner [22].

Several studies have been conducted to address the optimization problems in recommendation algorithms. In terms of data sparsity, Alhijawi et al. [23] proposed a collaborative filtering method based on demographic statistics, which can predict missing score data by learning user attribute characteristics. Yu et al. [24] suggested using singular value decomposition to process the original data matrix, improving data sparsity through decomposition and dimensionality reduction. Goldberg et al. [25] examined principal component analysis to preprocess data, analyzing and selecting essential data components to represent most variables. Huang et al. [26] studied graph-based methods to construct an information matrix by using the transitivity of user preferences to improve data sparsity. To tackle the cold start problem, Li et al. [27] utilized social tags to alleviate the cold start issue by adding labels to the prediction condition. Fayyaz et al. [28] adopted a heuristic similarity calculation method when calculating similarity, which alleviated the cold start problem for new users. Zhao et al. [29] put forward a functional matrix decomposition model that combines decision trees and matrix decomposition. By learning user preferences, the model selects items related to users and scores them using the matrix decomposition method. To address the problem of large data volumes, researchers such as Paun et al. [30] proposed the famous SlopeOne algorithm, which simplifies the regression function in collaborative filtering, significantly reducing the calculation time and storage requirements while achieving a recommendation effect equal to or even better than the collaborative filtering algorithm. Kataria & Batra [31] studied the co-clustering method, simultaneously clustering users and items, reducing computational complexity by looking for neighbors.

Despite the progress made in recommendation algorithms, there remain limitations in existing approaches that necessitate further research. For example, many traditional recommendation algorithms struggle with scalability and computational efficiency, particularly when dealing with large-scale datasets [32]. Additionally, the cold start problem remains a challenge in cases where there is insufficient data on new users or items [33,34]. Moreover, existing recommendation algorithms often do not adequately address the diversity of recommendations, potentially leading to filter bubbles where users are only exposed to content similar to their past preferences [35].

Frequent item set mining is a vital technique in recommender systems, aiming to discover sets of items that frequently appear together in user interactions. This process uncovers meaningful associations, enhancing the generation of personalized recommendations. In recommender systems, frequent item set mining addresses challenges like data sparsity and the “cold start” problem.

In this study, we propose an approach that addresses some of the identified problems in the literature by developing a novel algorithm for efficiently identifying frequent item sets in commodity datasets and utilizing them for commodity recommendations. Our proposed algorithm aims to filter out invalid commodity information and obtain valuable commodity data, potentially addressing issues such as data sparsity, cold start, and scalability. By incorporating this approach into the recommendation system, we hope to improve the overall performance and effectiveness of the system, providing more accurate and diverse recommendations for users.

## 3 Fusion recommendation algorithm based on frequent item set mining

This paper employs a data mining approach, treating the commodity set as the dataset for analysis. The commodity dataset will undergo space compression to identify frequent commodity sets. The recommendation order of commodities will be intelligently arranged through the calculation of user-commodity interest, prioritizing the recommendation of items with the highest rankings.

### 3.1 Existing problems of fusion recommendation algorithm based on frequent item set mining

Frequent item set mining, integrated into recommendation algorithms, explores correlations among items within vast datasets, emphasizing a set minimum expected support degree. However, issues arise with the increasing count of candidate frequent item sets, diminishing overall search efficiency. Addressing these challenges without compromising the profound relationships between recommended items is pivotal for algorithmic enhancement. Specifically, drawbacks include the generation of unnecessary rules from large datasets, lower recommendation accuracy and extended runtime of single-layer mining algorithms, and an inability to fully explore deep connections between users and items.

### 3.2 Proposed fusion recommendation algorithm

This paper proposes a fusion recommendation algorithm based on the frequent item set mining to solve the problems we found in the above research. This method uses frequent item set mining to filter product information and reduce the vast amount of product data. By retaining the frequent product dataset and calculating the product interest degree, the user's preferences can be ranked. The proposed algorithm is used for data mining and, compared to other data mining algorithms, reduces the number of frequent product datasets and weighted frequent item sets, thereby decreasing the time required for algorithm operation.

#### 3.2.1 Algorithm design

**Fig. 1:**
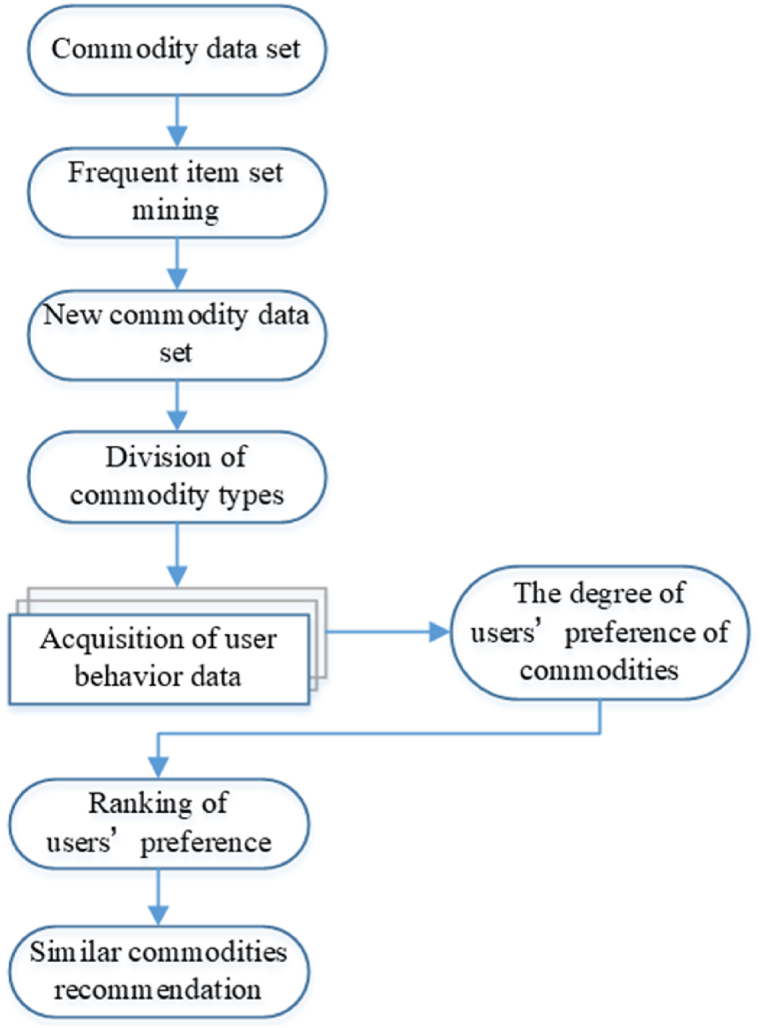
Information filtering graph based on the proposed algorithm.

The recommendation based on the proposed algorithm can be divided into three parts. The first part is to filter the information of the commodity data set based on the algorithm, find the frequent commodity data set and filter out the useless commodity data. The second part is mainly to divide the new commodity set into commodity data types and then calculate the commodity interest degree of the commodity subcategory combined with the user behaviour data, guess the user's liking degree of the commodity and sort it. The third part is the calculation method used to recommend the same type of products, and the recommendation results are given and displayed. The whole process and design content are shown in Fig. 1 below.

The algorithm proposed in this paper identifies frequent item sets in the commodity dataset and uses these sets for product recommendations. The primary goal is to filter out irrelevant commodity information and obtain valuable product data. The algorithm consists of the following steps:

Initialization: Generate a candidate 1-item set from each item in the commodity set. Calculate the top K item sets with the highest expected weighted support from the candidate 1-item set, denoted as TKWFIS1. Verify if the candidate frequent 1-item set is a weighted frequent 1-item set according to the definition. If so, add the result to the set WFIS1, denoted as a weighted frequent 1-item set.

Generation of 2-item sets: Set K = 2 when the frequent 1-item set WFIS1 is not empty. Link the frequent 1-item set WFIS1 and TKWFIS1 to generate the candidate weighted frequent 2-item set CWFIS2. Verify if the candidate frequent 1-item set is a weighted frequent 1-item set according to the definition. If so, add the result to the set WFIS2, and record it as a weighted frequent 2-item set.

Generation of K-item sets: Increment K by 1 (K=K + 1) and repeat Step 2.

Termination: Continue the process until WFIS is an empty set. The final result is the union of all weighted frequent item sets: WFIS=WFIS1 ∪ WFIS2 ∪ … ∪ WFISk.

Finally, the size of the weighted frequent item set is obtained according to the algorithm. The resulting weighted frequent item set in WFIS forms a frequent item set, and other unmatched items are automatically filtered out. The new commodity set obtained needs to be sorted according to the user's interest degree, mainly involving the calculation of user-commodity interest degree and the recommended rules of similar commodities. The following two sections will detail the specific calculation rules and contents.

#### 3.2.2 User-commodity interest ranking calculation

The proposed algorithm filters the commodity data set and obtains a new frequent commodity set in which user-commodity interest is calculated. The first is to conduct behavioral data analysis of users, based on the historical behaviour of users, comprehensive calculation of commodity interest. By dividing user behaviours, this paper only considers the following five user behaviours in the calculation: browsing goods(view), adding to favorite goods(fav), adding to shopping cart(cart), buying goods(buy) and evaluating goods(eval). Times indicates the period, and r indicates the weight of each behaviour of the user. The main operational steps of attribute weight assignment are: 1) Identification of relevant attributes. 2) Normalization of attribute contribution. 3) Parameter tuning. 4) Validate the assigned weights' appropriateness by using statistical methods. 5) Introduction of a randomization factor in the weight assignment to increase the test results.

Then, the user's interest degree for the commodity is F(u,j) = w(u, i), Ni), which represents the set of all commodity categories. When IEN(3) calculates the user's interest in the commodity, the behaviour not generated by the user is ignored (denoted as 0).

W(i) represents the user u's liking for Class i commodities. After calculating W(u,i) well, user preferences can be sorted, such as W(cosmetics) > W(toiletries) > W(snacks), so that users can roughly calculate the type of products they like, and the product description at the bottom of the list is not popular with users.

The dynamic update method is also adopted when calculating the degree of interest in goods because users' behavioral data include browsing and collection records. In order to better and accurately capture users' interest, the commodity category (commodity subcategory) in the order is detected. When the system detects a new item category in the user's order, it considers whether to add that item to the recommendation list. The recommendation system incorporates a dynamic update mechanism primarily triggered by the generation of new order numbers. This mechanism ensures that the system stays current with user preferences and evolving trends. When a new order is detected, the system follows these steps:(1) Identification of new item category; (2) Interest degree calculation; (3) Preference ranking update; (4) Continuous monitoring; (5) Dynamic update for real-time recommendations.

The concrete implementation can be divided into the following steps.

**Fig. 2:**
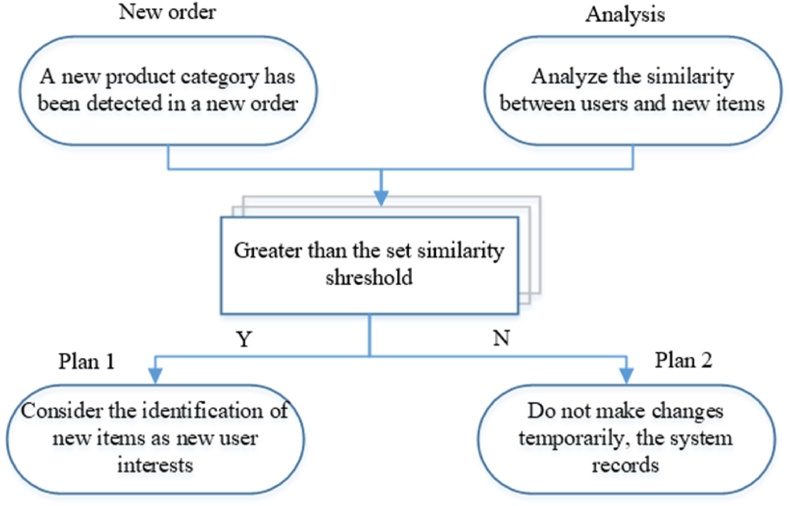
New product identification flow chart.

As shown in Fig. 2, once the system detects the presence of a new item category in the user's purchase order, the system records it. Then, the user's interest in the product should be calculated using user's interest degree for a commodity, mentioned in this section. If the obtained interest value is greater than the set interest reading value, it will be judged that the user's liking for the item is relatively high. Therefore, it is considered to add the new type of item to the commodity candidate set to facilitate the subsequent sorting of the commodity. The user's liking ranking of the commodity may change. If the calculated interest value does not exceed the set interest reading value, the default data will not be updated, and the appearance of new items will not impact the recommendation result temporarily. However, the system will record the appearance of the new item. If the purchase record of this kind of item occurs again, the final impact value should be considered comprehensively. Once the interest threshold is exceeded, the new type of item will be added to the commodity candidate set for the next calculation step.

In the recommendation technology based on deep learning, there have been studies on using neural network algorithms to capture the change in users' dynamic preferences for a real-time recommendation. Although recommendation algorithms based on deep learning have made promising breakthroughs in data sparsity, cold start and interpretability, and such recommendation systems can capture the changes of users' interests in real-time, such algorithms are often complex in structure calculation, challenging to implement, and not applicable to all backgrounds. This study adopts the most straightforward polling mechanism to achieve dynamic recommendations. The advantages of the polling mechanism include the following.

(1) The idea is simple. The structure is easy to realize.
(2) It can be agile to capture the user's change of interest in the new project.
(3) Users' historical data and purchase records are used as verification data to ensure the data's authenticity, reliability and interpretation.

#### 3.2.3 Similar product recommendation rules

**Fig. 3:**
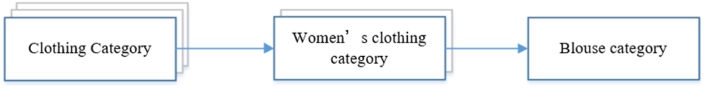
Commodity classification diagram.

In order to classify commodities, the system classifies all commodities into three levels, which are classified into major (level 1), middle (level 2) and small (level 3) categories. Commodity based collaborative filtering algorithm is adopted to calculate the similarity of similar commodities. Commodities are divided into three categories, such as clothing, women's clothing and jacket, during the calculation of similarity between commodities. Only the similarity of goods between the same subclasses is calculated. At the same time, each type of commodity is given the characteristic value and the corresponding weight value through the calculation of the similarity between commodities. The whole process and design content are shown in Fig. 3 below.

When calculating the similarity F between commodity a and commodity b, the characteristics of commodity subclasses are considered. Commodities characteristic and attribute mapping said form such as: F (a, b) = (sim: r). (top) = F (“ brand ": 0.2,” price ": 0.3,” color ": 0.2,” style ": 0.2,” size ": 0.1]. As shown in Table 1.

**Table 1:** Weight table for similarity calculation.

Eigenvalue a	r brand	r price	r color	r style	r size
Weight a	0.2	0.3	0.2	0.2	0.1
Weight b	0.2	0.4	0.1	03	0.1

The representation of these attributes may vary or be inconsistent across products or categories. For example, in the context of apparel, “style” may refer to design elements such as casual, formal or sporty. In the context of electronic devices, “style” may be associated with elements such as sleek, modern, or vintage design. Therefore, we propose standardized definitions for each attribute in Table 1 to ensure that their meanings are consistent across all product categories. This eliminates ambiguity in the attribute representation.

**Fig. 4:**
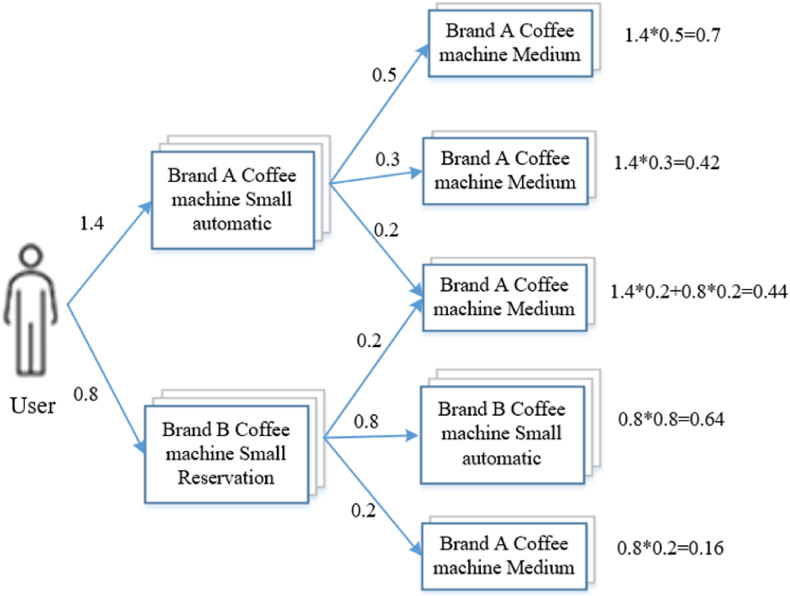
The method of calculating the user's interest in the commodity.

After calculating the user-commodity interest degree using Fig. 4, this section mainly calculates the similarity between similar commodities. Assume that users have generated historical data for “Brand A coffee machine Small automatic” and “Brand B coffee Machine Small Reservation” in household appliances, calculate users' interest in these products according to the format (brand, specification and function), assume that the calculated interest values are 1.4 and 0.8, and then calculate the similarity of similar products on this basis. A diagram of similar products that should be recommended to the user is shown in Fig. 4 below.

Through similarity calculations, products with diminishing relevance or user interest are effectively identified. Unmatched items are automatically filtered out during the process, ensuring that the recommendation list remains pertinent. Through the similarity calculation of similar products, according to the calculation results from large to small order, we should recommend the commodity “A brand coffee machine medium”, “B brand coffee machine small automatic”, and “coffee machine automatic” in turn. Calculating the similarity of similar commodities can effectively capture the keyword information searched by users. In the recommendation process of the existing system, it is necessary to describe the keyword of the commodity, and different keywords will present different commodity information. Therefore, in the recommendation process, it is also preferred to recommend the commodity with high similarity in the calculation result.

## 4 Experimental results and analysis

### 4.1 Experimental data

#### 4.1.1 Dataset characteristics

In this experiment, two data sets are selected for the experiment. Namely commodity data set TD and real-world commodity data set R. The characteristics of the data set are shown in Table 2 below, where |D| is all commodity sets included in the data set, |I| is all users included in the data set, and Avg is the average number of commodities each user is interested in.

**Table 2:** Dataset characteristics.

Dataset	|D|	|I|	Avg
TD	100,000	870	10.1
R	88,160	16,470	10.3

#### 4.1.2 Dataset sources

The digital transactions dataset (DT) is synthetic and generated for experimental purposes, simulating user-item interactions in the context of data mining and recommender systems [36]. This data set simulates the user-item interactions and provides a controlled environment for testing our proposed algorithm.

The real-world commodity data set (R) is sourced from an e-commerce dataset, which contains detailed information about user-item interactions, including user browsing, collecting, shopping cart, purchase, and evaluation of basic data [37]. This dataset provides a realistic scenario for evaluating the effectiveness of our proposed algorithm and has been used in various research studies in the field of recommendation systems.

By using both synthetic and real-world datasets, we aim to demonstrate the performance of our proposed algorithm in different contexts, providing a comprehensive evaluation of its efficiency and effectiveness.

In addition to the algorithm proposed in this research, the uapriori algorithm [38] and the hewi-uapriori algorithm [39] were also selected for comparison. The experiment is divided into two directions, including the time efficiency contrast of the algorithm and the comparison of the number of frequent items. For part of the data set, it is needed to explain two things. First, the synthetic commodity data set td and the actual commodity data set r contain data from users and goods, including the user's browsing, collecting, shopping cart, purchase, and evaluation of basic data. In the second, the weighted frequency of the uapriori algorithm is called wfis1, the weighted frequency of the hewi-uapriori algorithm is called wfis2, and the weighted frequency number of times for the algorithm proposed in this article is called wfis3.

Before analyzing the performance of the algorithm, we need to make clear two points: the weight of the various behaviors represented by the user and the characteristics of the small class of the goods, which is to calculate the similarity of the goods in order to follow up and to get the user's preference for the goods. In order to avoid the effect of the characteristics weight of the product characteristics on the time efficiency of the algorithm, we used 20 groups of different weights in the experimental process, and the characteristics of the goods were analyzed. The results of the experiment were the average of each experiment. These results demonstrate the effectiveness of the proposed algorithm in comparison to the uapriori and hewi-uapriori algorithms in terms of time efficiency and the number of frequent items identified.

### 4.2 Time efficiency comparison

#### 4.2.1 Experimental setup

The first set of experiments focuses on the running time of the three algorithms on both datasets. The minimum expected weighted support varies, allowing the observation of algorithm behaviour under consistent commodity attribute weights.

#### 4.2.2 Results and analysis

**Fig. 5:**
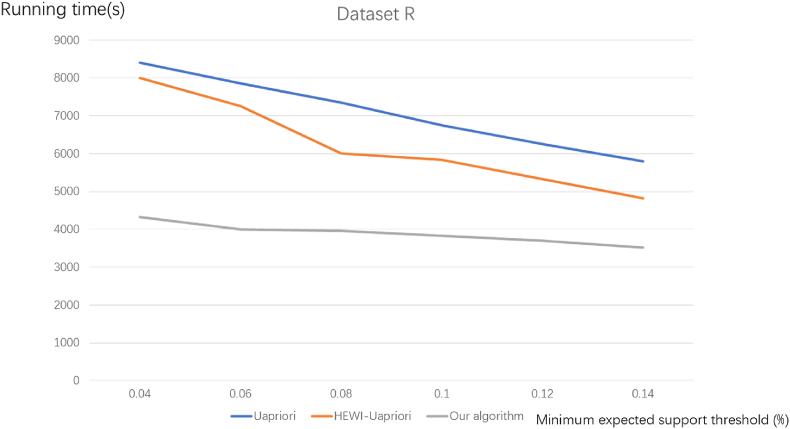
Comparison of algorithm running time for commodity dataset R.

**Fig. 6:**
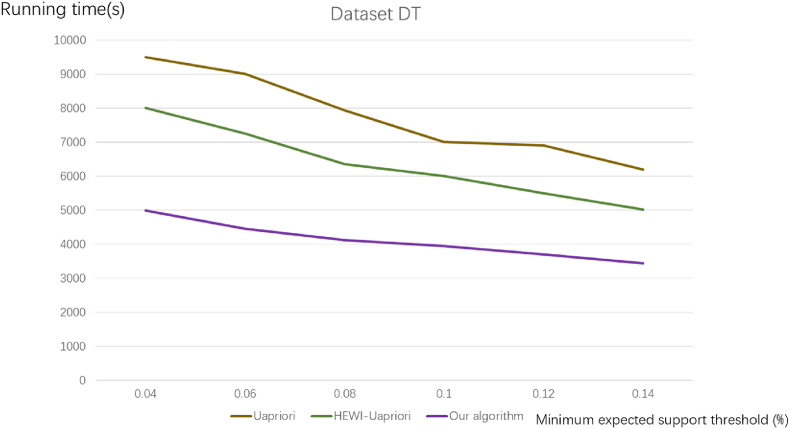
Comparison of algorithm running time for commodity dataset DT.

Fig. 5, Fig. 6 illustrate the running time comparison for commodity datasets R and TD, respectively.

It can be seen from Fig. 5, Fig. 6 that the running time of this algorithm decreases with the increase of the minimum expected weighted support when the weight of commodity attribute features is consistent. Comparative analysis with two other algorithms reveals a harmonious trend, aligning with our proposed algorithm's behaviour. Nevertheless, a distinctive aspect is observed in the impact of varying minimum expected support reading values on the algorithms' running time. The experimental diagrams distinctly showcase that our proposed algorithm exhibits the shortest running time. In the realm of frequent item set mining, the conventional approach necessitates repeated verification of the database and item set during candidate frequent item set generation. In contrast, the proposed algorithm minimizes the redundant verification of weighted frequent candidate sets, resulting in significant time savings. These compelling experimental outcomes robustly affirm the superiority of our algorithm over the other two selected algorithms. Therefore, it can be concluded that the algorithm proposed in this paper is more efficient and faster.

To further validate the performance of this paper's method, the proposed algorithm is compared with the mainstream SOTA algorithm under two metrics, namely, AUC (area under ROC curve) and MEA (mean absolute error). The SOTA algorithm is taken from the literatures [[40], [41], [42]], and [43], respectively. The test results on the two datasets are shown in Table 3.

**Table 3:** Performance comparison of the proposed algorithm with other SOTA algorithms on 2 datasets.

Algorithm	TD		R	
	AUC	MEA	AUC	MEA
Literature [40]	0.8002	0.7591	0.8797	0.8611
Literature [41]	0.8085	0.7426	0.8814	0.8241
Literature [42]	0.8034	0.6949	0.8823	0.7466
Literature [43]	0.8103	0.6776	0.8835	0.7534
Proposed	0.8215	0.6311	0.8986	0.7236

It can be seen from Table 3 that the proposed algorithm has a large improvement in MEA compared to other SOTA algorithms on two datasets. Meanwhile, the AUC of this paper's algorithm is the highest compared to the other four algorithms, which indicates that the proposed model has the best performance.

## 5 Conclusion

In this study, we proposed a fusion recommendation algorithm based on frequent item set mining to improve recommendation efficiency and accuracy in e-commerce platforms. The algorithm was evaluated using two data sets, TD and R, and compared with the uapriori and hewi-uapriori algorithms in terms of time efficiency and the number of frequent items.

Our experimental results demonstrated that the proposed algorithm outperformed the other two algorithms in terms of running time, showing that it is more efficient and faster. This improvement can be attributed to the reduction of repeated verification of the weighted frequent candidate sets, which saves a significant amount of time.

However, there are some limitations to our research. Firstly, the algorithm's performance may be affected by the choice of weight values for different product features, which can influence the time efficiency of the algorithm. Although we used 20 groups of different weights in the experimental process, more comprehensive analysis and optimization may be needed to determine the optimal weight values. Secondly, our experiments were conducted on two data sets with specific characteristics. Further research is required to validate the algorithm's performance on a wider variety of data sets and in different e-commerce platforms.

In conclusion, the fusion recommendation algorithm based on frequent item set mining presented in this study shows promising results in improving recommendation efficiency and accuracy. However, future work should focus on addressing the mentioned limitations to enhance the algorithm's applicability and performance in diverse e-commerce scenarios.

## Data availability statement

The labeled dataset used to support the findings of this study are available from the corresponding author upon request.

## CRediT authorship contribution statement

**Lifeng Kang:** Visualization, Resources, Formal analysis. **Yankun Wang:** Writing – review & editing, Visualization, Validation, Methodology.

## Declaration of competing interest

The authors declare the following financial interests/personal relationships which may be considered as potential competing interests: Wang Yankun reports was provided by Jiaozuo Normal College. Wang Yankun reports a relationship with Jiaozuo Normal College that includes: employment. If there are other authors, they declare that they have no known competing financial interests or personal relationships that could have appeared to influence the work reported in this paper.
